# Exposure to solar ultraviolet radiation establishes a novel immune suppressive lipidome in skin-draining lymph nodes

**DOI:** 10.3389/fimmu.2022.1045731

**Published:** 2023-01-20

**Authors:** Benita C. Y. Tse, Angela L. Ferguson, Yen Chin Koay, Georges E. Grau, Anthony S. Don, Scott N. Byrne

**Affiliations:** ^1^ The University of Sydney, School of Medical Sciences, Faculty of Medicine and Health, Sydney, NSW, Australia; ^2^ Heart Research Institute, The University of Sydney, Sydney, NSW, Australia; ^3^ Westmead Institute for Medical Research, Centre for Immunology and Allergy Research, Sydney, NSW, Australia

**Keywords:** immune regulation, immune suppression, lipids, ultraviolet radiation, mass spectrometry, extracellular vesicles

## Abstract

The ability of ultraviolet radiation to suppress the immune system is thought to be central to both its beneficial (protection from autoimmunity) and detrimental (carcinogenic) effects. Previous work revealed a key role for lipids particularly platelet-activating factor and sphingosine-1-phosphate in mediating UV-induced immune suppression. We therefore hypothesized that there may be other UV-induced lipids that have immune regulatory roles. To assess this, mice were exposed to an immune suppressive dose of solar-simulated UV (8 J/cm^2^). Lipidomic analysis identified 6 lipids (2 acylcarnitines, 2 neutral lipids, and 2 phospholipids) with significantly increased levels in the skin-draining lymph nodes of UV-irradiated mice. Imaging mass spectrometry of the lipids in combination with imaging mass cytometry identification of lymph node cell subsets indicated a preferential location of UV-induced lipids to T cell areas. *In vitro* co-culture of skin-draining lymph node lipids with lymphocytes showed that lipids derived from UV-exposed mice have no effect on T cell activation but significantly inhibited T cell proliferation, indicating that the lipids play an immune regulatory role. These studies are important first steps in identifying novel lipids that contribute to UV-mediated immune suppression.

## 1 Introduction

Exposure to the ultraviolet (UV) radiation in sunlight suppresses the local cutaneous immune response and is a major risk factor for the development of UV-induced skin cancers (1). The formation of pyrimidine dimers (2, 3) and production of *cis*-urocanic acid (4) within the skin following UV exposure are well-established molecular signals responsible for local immune suppression. However, UV is also capable of causing systemic immune suppression which is associated with protection from several autoimmune diseases (5). The suppressive signal generated in UV irradiated skin is transmitted to local draining lymph nodes by migrating Langerhans cells (LC) (6, 7) and mast cells (8). Recently arrived LCs interact with Natural Killer (NK) T cells to suppress the immune response in an IL-4-dependent manner (7) while IL-10-producing mast cells (9) interact with follicular B cells (8). Ultimately, these early cellular events lead to the generation of antigen-specific, long-lived UV-Tregs (10, 11) and UV-Bregs (12–15). The skin-draining lymph nodes are therefore a key anatomical site for UV-suppression of immunity.

Recently we showed that a single dose of solar-simulated UV altered lymphocyte recirculation between the skin-draining lymph nodes and the peripheral blood (16). This phenomenon was due to UV increasing the amount of the chemotactic lipid, sphingosine-1-phospate (S1P), in the lymph nodes. The increase in S1P led to a downregulation of sphingosine-1-phospate receptor 1 (S1P_1_) receptors and a sequestration of naïve and central memory T cells in the lymph nodes. This provided the first evidence that UV can modulate the immune system by manipulating lipids in the skin-draining lymph nodes.

There is a growing recognition for the role of bioactive lipids and lipid metabolism in modulating the immune response. Lipid oxidation for example, is required for regulatory T cell proliferation (17–19) and the generation of memory T cells (20). In addition, bioactive lipids such as lysophosphatidylserine suppress CD4^+^ T cell activation and proliferation by inhibiting IL-2 production and the upregulation of early activation markers such as CD69 (21, 22). Binding of lysophosphatidylserine also suppresses the generation of regulatory T cells (21), in which IL-2 inhibition is likely similarly involved. Indeed, UV radiation alters lipid in the skin by increasing production of sphingolipids, prostanoids and hydroxy fatty acids whilst reducing production of free fatty acids and triglycerides (23–25). Of particular importance, UV radiation of the skin triggers the release of the immunosuppressive lipid, platelet-activating factor (PAF) and its analogs. Activation of PAF receptors on dermal mast cells and Langerhans cells initiates their migration to the skin-draining lymph node (26, 27). Antagonism of PAF receptors and/or PAF-receptor deficiency attenuates UVB-induced systemic (28, 29), but not local immune suppression (30).

In light of our finding that UV upregulates S1P, in addition to other immune suppressive lipids such as PAF in the skin (31, 32), we hypothesized that exposure to UV may activate other immune modulating lipids in the skin-draining lymph nodes. In this study, we used non-targeted lipidomics to identify six novel UV-induced lipids in the skin draining lymph nodes. Using a combination of imaging mass spectrometry and cytometry of the nodes we were able to reveal that the upregulated lipids were preferentially expressed in the T cell areas following UV exposure. Finally, when the lipids induced by UV were isolated from the skin-draining lymph nodes and incubated with lymphocytes, they suppressed the proliferation of T cells. Thus, ultraviolet radiation induces immunosuppressive lipids where T cell activation occurs – in the skin-draining lymph nodes.

## 2 Materials and methods

### 2.1 Animals and UV radiation

Female C57BL/6 mice (Australian BioResources Ltd, Moss Vale, Australia) aged 7-10 weeks were housed at 22°C on a 12 h light-dark cycle with free access to water, and chow (Specialty Feeds, WA) provided ad libitum. The fluorescent lights used in the animal house did not emit any UV radiation. All mice were shaved and housed together and provided with sufficient nesting material for them to self-regulate their body temperature. Mice were shaved on the back and exposed to a single, immune suppressive 8 J/cm^2^ dose of solar-simulated UV generated with a 1000 W xenon arc solar simulator (Oriel, Stratford, CT) with an output of 8% UVB and 92% UVA. Full details on the UV spectra has been published by us previously (33). In female C57BL/6 mice, a solar simulated UV dose of 3.64J/cm^2^ is the minimum required to induce a statistically significant increase in skin thickness (the minimal edematous dose; MEdD) (33). Unlike humans, mice don’t make a readily detectable erythemal response to UV, and so the MEdD is used as a surrogate for the minimal erythemal dose (MED). Thus, the dose used is 2.2MEdD which translates to approximately 15min of midday summer sun in Sydney, Australia. Control mice were shaved, sham-irradiated and co-housed with irradiated mice. The animal experiments were approved by the University of Sydney Animal Ethics committee (#1352).

### 2.2 Imaging mass cytometry

Skin-draining (inguinal) lymph nodes were collected 24 hours post-UV radiation and freshly frozen in embedding media containing 5% v/v carboxymethylcellulose and 10% v/v gelatin (both Sigma, St. Louis, USA) which is optimal for mass spectrometry imaging, generating significantly less background than OCT (34). 10 µm sections were fixed in 100% cold acetone for 10 minutes. For staining, the slide was rehydrated, blocked using an avidin-biotin blocking kit (Life Technologies, Carlsbad, USA) and 10% mouse serum containing anti-CD16/32 antibody (50 µg/mL, clone 93, Biolegend, San Diego, USA). 50 μL of a master mix containing anti-mouse CD45-FITC, CD35-biotin, CD62L-APC and CD11b-PE in 2% mouse serum was added and stained at 22°C for 2 hours. The CD45-FITC antibody was used to provide visual confirmation that the staining process was successful. The use of CD35-biotin, CD62L-APC and CD11b-PE primary antibodies allowed for amplification of weaker antibody stains with a secondary metal-conjugated antibody and ensured that all our markers of interest could be included in the panel. Antibody clones and concentrations are stated in **Table 1**. The slide was then washed in Tris-buffered Saline and Tween (TBST; all from Sigma). A second master mix containing metal-conjugated antibodies was added to each section and incubated overnight at 4°C. The next morning, the slides were washed and fixed for 9 min using formalin (Fronine, Riverstone, Australia). Following fixation, the slide was washed in TBST and stained with iridium DNA intercalator for 30 min at 22°C. The slide was again washed first with TBST and then in distilled water. The slide was dried at 22°C and imaged within one week of staining. Imaging was conducted using the Hyperion Imaging System coupled to a Helios (Fluidgm, South San Francisco, USA).

**Table 1:** List of antibodies used for imaging mass cytometry.

Fluorochrome/Metal	Antibody	Clone	Concentration	Source
FITC	CD45	30-F11	5 µg/mL	BD
Biotin	CD35	8C12	1.25 µg/mL	BD
APC	CD62L	MEL-14	2 µg/mL	eBioscience
PE	CD11b	M1/70	2 µg/mL	Biolegend
Structural cell markers				
115In	Lyve1	Polyclonal	4 µg/mL	R&D
160Gd	Podoplanin	8.1.1	4 µg/mL	Biolegend
165Ho	CD31	390	4 µg/mL	BD
Pan-immune marker				
174Yb	anti-FITC/FITC-CD45	FIT-22	4 µg/mL	Biolegend
Myeloid markers				
142Nd	CD11c	N418	8 µg/mL	Biolegend
144Nd	Ly6G	1A8	2 µg/mL	Biolegend
146Nd	CD207 (Langerin)	4C7	4 µg/mL	Biolegend
154Sm	CD169	3D6.112	4 µg/mL	Biolegend
156Gd	Anti-PE/PE-CD11b	PE001	4 µg/mL	Biolegend
161Dy	F4/80	BM8	3 µg/mL	Biolegend
163Dy	CD64	X54-5/7.1	2 µg/mL	Biolegend
170Er	Anti-biotin/biotin-CD35	1D4-C5	1 in 200	DVS/Fluidgm
176Yb	Ly6C	HK1.4	2 µg/mL	Biolegend
T cell markers				
152Sm	CD3e	145-2C11	4 µg/mL	Biolegend
153Eu	CD4	RM4-5	1.8 µg/mL	Biolegend
168Er	CD8a	53-6.7	4 µg/mL	Biolegend
145Nd	CD69	H1.2F3	4 µg/mL	Biolegend
171Yb	CD44	IM7	2 µg/mL	BD
162Dy	Anti-APC/APC-CD62L	APC003	1 in 100	DVS/Fluidgm
158Gd	FoxP3	FJK-16s	1 in 200	DVS/Fluidgm
B cell markers				
149Sm	CD19	6D5	2 µg/mL	Biolegend
150Nd	IA-IE	M5/114.15.2	2 µg/mL	Biolegend
159Tb	B220	RA3-6B2	2 µg/mL	Biolegend
Pan-nuclei marker				
191Ir/193Ir	DNA intercalator		1.25 ng/mL	DVS/Fluidgm

### 2.3 Lipid extraction

Lipids were extracted from skin-draining lymph nodes as previously described (16). For lipidomic studies, 50 µL of pooled internal standards (all from Avanti Polar Lipids, Alabaster, USA) containing 20 µM C12 (18:1/12:0) glucosyl (β) ceramide (C12 GluCer), 20 µM C17 (18:1/17:0) ceramide (C17 Cer) and 4 µM of C17 (17:1) sphingosine (C17 Sph) were spiked into the extraction solution along with the lymph nodes. For T cell functional studies, no internal standards were added.

### 2.4 Non-targeted mass spectrometry

5 µL of lipid sample was injected into ThermoFisher Scientific Vanquish Ultra-High Pressure Liquid Chromatography (UHPLC) system coupled to a Waters Acquity UPLC C18 column (1.7 µm pore size, 2.1 x 100 mm). The HPLC operated with gradients of solvent A (60% acetonitrile, 40% water, 0.1% formic acid (Sigma) and 10 mM ammonium formate (Sigma)) and solvent B (90% isopropanol, 10% acetonitrile, 0.1% formic acid and 10 mM ammonium formate) (Organic solvents were from Fisher Chemical, New Hampshire, USA) (**Table 2**).

**Table 2:** LC gradients and flow-rate.

Time (min)	Flow-rate (mL/min)	% Solvent B
0.0	0.280	30
3.0	0.280	30
4.5	0.280	43
5.5	0.280	55
8.0	0.280	65
13.0	0.280	85
14.0	0.280	100
20.0	0.280	100
20.2	0.360	30
24.8	0.360	30
25.0	0.280	30

The lipid scan was conducted using a Thermo Fisher Scientific Q Exactive HF-X Hybrid Quadrupole-Orbitrap™ mass spectrometer in full scan/data-dependent MS/MS (ddMS^2^) mode between *m/z* 220 and 1600. These scans were conducted in both positive and negative ion mode to ensure all classes of lipids are captured. Analysis of the peaks was conducted using Compound Discoverer (Thermo Fisher Scientific, Waltham, USA).

Molecules of interest were fragmented either by adding the lipids of interest onto the inclusion list, or by using a targeted- selected ion monitoring followed by ddMS^2^ in both positive and negative ion mode. The fragmentation pattern was analyzed in LipidSearch 4.0 (Thermo Fisher) to identify the molecules. The identification was considered correct if: 1) An A or B grade was obtained in LipidSearch (grading indicates confidence in identifying the fatty acids and lipid group); 2) fragments were consistent with identification (e.g. fragments of headgroups and fatty acids were found); and 3) fragmentation and identification was consistent across 3 individual experiments.

### 2.5 Mass spectrometry imaging

The lymph nodes were cryosectioned at 10 µm thickness and mounted onto indium tin oxide (ITO) slides (Bruker Daltonics, Billerica, USA). The slide was prepared for matrix-assisted laser desorption/ionization (MALDI) mass spectrometry imaging following a previously established method (35) to sublimate recrystallized 2, 5-dihydroxybenzoic acid (DHB) (Sigma) onto the ITO slide. The slide was then loaded into the UltrafleXtreme (Bruker) mass spectrometer. Elemental red phosphorus (Sigma) was used to calibrate the instrument. The sections were then ablated at 15 µm raster width (i.e. 15 µm spatial resolution) and full scan analyzed at *m/z* 380-1800. The peaks and images were analyzed on SCiLS lab (Bruker).

### 2.6 Primary lymph node cell culture with lipids

Naïve skin-draining lymph nodes were collected inside biosafety cabinets under sterile conditions before being dissociated with 25 gauge needles (Terumo, Shibuya City, Japan) and cells passed through a cell strainer (Miltenyi, Bergisch Gladbach, Germany) to obtain single cell suspensions. Cells were then counted using a haemocytometer (Livingstone, Toronto, Canada) and viability assessed using trypan blue (Life Technologies, Carlsbad, USA).

For proliferation assays, cells were prepared for carboxyfluorescein succinimidyl ester (CFSE; Thermo Fisher) staining by washing once in RPMI 1640 medium (Life Technologies) then resuspending in pre-warmed RPMI to a concentration of 5 x 10^7^ cells/mL. CFSE was added to the cells at a 1 in 1000 dilution (final concentration 5µM). The cells were immediately inverted and placed in a 37°C incubator for 10 minutes. Cells were inverted every 3-4 minutes and the staining stopped with the addition of cold complete RPMI (RPMI with 10% charcoal-stripped FCS (Thermo Fisher), 0.05 μM 2-mercaptoethanol (Sigma) and 1% penicillin-streptomycin (Invitrogen, Waltham, USA)) at 4 times the initial volume. Cells were washed twice more with complete RPMI.

For T cell activation assays, non-CFSE-labelled cells were seeded into U-bottom 96-well plates (Corning, New York, USA) at 2 x 10^5^ cells/well. For T cell proliferation assays, CFSE-labelled cells were seeded into the plates at 5 x 10^5^ cells/well. Dried lipid preparations were reconstituted in complete RPMI and added to cells at a ratio of one lymph node amount of lipids to one lymph node number of cells as previously described (16). A final concentration of 0.27 μg/mL of anti-CD3 (clone 1452c11) and 0.17 μg/mL of anti-CD28 (Biolegend, clone 37.51) antibodies were added to the cells. The plates were incubated at 37°C with the activation plate analyzed after 24 hours and proliferation analyzed at 72 hours.

### 2.7 Large extracellular vesicle isolation and analysis

To assess large extracellular vesicles (LEVs), 6 hours post-UV (or sham-UV) treatment, skin-draining lymph nodes were collected in double filtered FACs buffer (phosphate-buffered saline (PBS) with 0.5% bovine serum albumin (BSA, Bovogen, Melbourne, Australia) and 0.4% ethylenediaminetetraacetic acid (EDTA)). Lymph node capsules were broken apart and a single-cell suspension achieved, retaining both the resulting cells and suspension. Lymph node cell viability was assessed by trypan blue staining and quantified using a haemocytometer. LEVs were harvested from all samples using multiple centrifugations: 1500x*g* for 15 minutes to remove cells, 13000x*g* for 2 minutes to remove cellular debris and platelets, and 18000x*g* for 60 minutes at 4°C to pellet LEVs. LEV pellets from each condition were then resuspended in a final volume of 100μl filtered PBS and kept at -80°C until they were prepared for flow cytometric analysis.

### 2.8 Flow cytometry staining

Cells were washed and pre-stained with Fixable Viability Dye eFluor 455UV (Thermo Fisher) and FcBlock (Biolegend) for 15 minutes at 22°C. Cells were then washed and 50 μL of primary antibody mix added (**Table 3**). Cells were stained in the dark at 4°C for 30 minutes. For the proliferation studies, cells were then washed, fixed in 50 μL of fixation buffer (Biolegend) for 20 minutes, washed and resuspended in FACs buffer ready for analysis. For the activation studies, cells were prepared for intracellular staining using a FoxP3/transcription factor staining buffer set (Thermo Fisher). Cells were fixed and permeabilized for 20 minutes at 22°C. Cells were then washed with permeabilisation buffer and resuspended in 50 μL of intracellular antibody mix. After 30 minutes of staining at 22°C, cells were washed again with permeabilization buffer and then resuspended in FACs buffer ready for analysis.

**Table 3:** List of flow cytometry antibodies.

Marker	Fluorochrome	Concentration	Clone	Company
Common to both lymph node cell panels				
CD16/32	Purified	5 µg/mL	93	Biolegend
Live/dead	eFluor 455UV	1 in 1000		Thermo Fisher
CD3ε	PE-CF594	1 µg/mL	145-2C11	BD
CD4	PerCP	1 µg/mL	RM4-5	Biolegend
Cell activation panel (24 hours)				
CD8α	FITC	2.5 µg/mL	5H10-1	Biolegend
CD62L	BV421	1 µg/mL	MEL-14	BD
CD44	BV786	0.2 µg/mL	IM7	BD
CD25	APC-eFluor780	2 µg/mL	PC61.5	eBioscience
FoxP3 (intracellular)	PE	1 in 50	150D	Biolegend
CD69	PE-Cy7	1 µg/mL	H1.2F3	Thermo Fisher
S1P_1_	APC	1 in 100	713412	R&D
Cell proliferation panel (72 hours)				
CD8a	APC-Cy7	1 µg/mL	53-6.7	Biolegend
CD11c	APC	2 µg/mL	N418	eBioscience
CD19	PE	1 µg/mL	eBio1D3	eBioscience
B220	BUV737	1 µg/mL	RA3-6B2	BD
I-A/I-E	BV510	2.5 µg/mL	M5/114.15.2	Biolegend

LEVs were stained with annexin-V AF488 (BD, Franklin Lakes, USA) and selected parent antibodies: T cells (CD4, Biolegend, clone RM4-5 or CD8, eBioscience, clone 53-6.7), B cells (CD19, eBioscience, clone 1D3), monocyte/macrophages (CD11b, Biolegend, clone M1/70), platelets (CD41, BD,clone MWReg30), endothelial cells (CD105, BD, clone MJ7/18), mast cells (CD117, Biolegend, clone ACK2) or keratinocytes (pan-keratin, CST, clone C11) for 25 minutes in binding buffer and enumerated on a BD LSRFortessa for 120 seconds at medium flow rate. Countbright, absolute counting beads (Invitrogen) were used as an internal standard to allow direct enumeration of EVs per microlitre of supernatant. LEVs were analyzed on a FSC vs SSC dot plot. To define the LEV gate, we used 0.22–1.34 μm latex beads (Nano Fluorescent Size Standard Kit, Yellow, Flow Cytometry Grade, Spherotech, Lake Forest, USA). Events falling within the LEV gate were then analyzed for parent-antibody and AnnexinV-AF488 fluorescence on a cytogram.

### 2.9 Statistics

For experiments with *n* values of greater than 6, comparisons between 2 groups were done by Student’s t tests. For experiments with *n* values of 6, a Shapiro-Wilks normality test was performed (α=0.05; p<0.05) and for normally-distributed data, a Student’s t test (comparing 2 groups) or ordinary one-way ANOVA with a Holm-Sidak multiple comparisons test (comparing more than 2 groups) were used. For groups that failed the Shapiro-Wilks normality test, and for experiments with *n* values less than 6 a non-parametric analysis using Mann-Whitney test (comparing 2 groups) or Kruskal-Wallis test with Dunn’s multiple comparisons (comparing more than 2 groups) were used. Data from individual mice was expressed as a fold change (normalized) to the mean of the No UV control group in each experiment. Thus the relative expression/frequency of the No UV group would be a value of 1, enabling different experimental repeats to be pooled.

## 3 Results

### 3.1 UV does not alter skin-draining lymph node architecture nor T cell distribution

**Figure 1:**
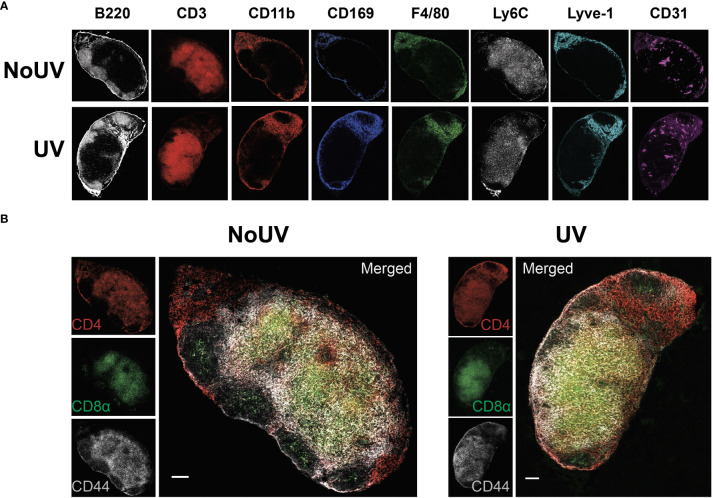
UV exposure does not alter skin-draining lymph node architecture. Skin-draining (inguinal) lymph nodes were collected from mice exposed (or not) to 8 J/cm^2^ UV 24 h after exposure. Lymph node sections were stained and analyzed by imaging mass cytometry. **(A)** Representative images of immune and structural markers. **(B)** Representative images of CD4^+^, CD8^+^, and CD44^+^ T cells. Scale bars represent 100 µm.

We have previously demonstrated that exposure to UV results in local skin-draining lymph node hypertrophy (8, 16). To determine if this UV-induced lymph node hypertrophy is associated with changes in lymph node architecture, we performed imaging mass cytometry assessing 24 immune and structural markers. UV-exposure did not change gross lymph node architecture, with B cell follicles (B220^+^), T cell areas (CD3^+^), macrophage areas (defined by varying expressions of CD11b, CD169 and F4/80), lymphatics (Lyve-1^+^) and high endothelial venules (CD31^+^) all appearing to be similar to control unirradiated lymph nodes (**Figure 1A**)

UV-irradiated skin-draining lymph nodes preferentially sequester naïve and central memory T cells (16). Imaging mass cytometry revealed that CD4^+^ and CD8^+^ T cell distribution was similar between unirradiated control and UV lymph nodes with both T cell subsets abundant in the cortical area and CD4^+^ T cells prominent in the interfollicular space closer to the lymphatic sinuses (**Figure 1B**). This is consistent with other studies demonstrating that CD4^+^ T cell localize with lymph node resident cDC2s close to the lymphatic sinus, whereas CD8^+^ T cells localize with the more centrally-located resident cDC1 population (36). In both groups there was a greater concentration of CD44^+^ memory T cells adjacent to the follicles. Hence, UV-induced lymph node hypertrophy is not associated with changes in lymph node architecture.

### 3.2 UV-radiation alters the skin-draining lymph node lipidome

**Figure 2:**
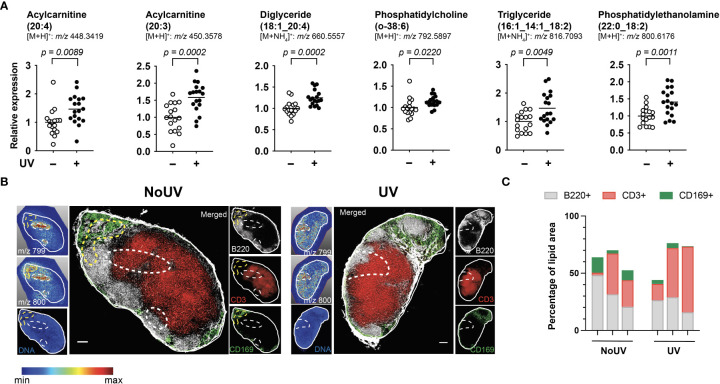
UV altered several lipids in the skin-draining lymph nodes with possible preferential localization to T cells areas. Skin-draining lymph nodes were collected from UV-irradiated and control mice 24 h after exposure. **(A)** Lipids extracted from skin-draining lymph nodes were analyzed by non-targeted mass spectrometry. Normalized relative expression of the lipids (acyl chain) across 3 independent UV experiments (each with *n* = 5-6 mice per group) are shown. Statistics were done by Student’s t test with mean shown. **(B)** Mass spectrometry imaging and imaging mass cytometry were conducted on serial sections of lymph nodes. Representative lymph nodes shown with expression of lipids dictated by the gradient and dotted lines indicating high lipid areas. Scale bar represents 100 µm. **(C)** Areas of high lipid were quantified for the presence of CD169, B220 and CD3 expression for lymph nodes from 6 individual mice (3 NoUV and 3 UV).

We next assessed if UV radiation of the skin alters lipids within the skin-draining lymph nodes. Lipids extracted from the skin-draining lymph nodes were analyzed using non-targeted mass spectrometry. Six identifiable lipids were significantly increased in the skin-draining lymph nodes following UV exposure (**Figure 2A**). These lipids were acylcarnitine (20:4) (neutral mass of 447.3348), acylcarnitine (20:3) (449.3505), diglyceride (18:1_20:4) (642.5221), phosphatidylcholine (o-38:6) (791.5823), triglyceride (16:1_14:1_18:2) (798.6743) and phosphatidylethanolamine (22:0_18:2) (799.6102). Thus the UV-altered lipids belonged to a variety of lipid classes.

We next determined whether the UV altered lipids shared similar anatomical distribution across the lymph node. Since lipids cannot be imaged by immunohistochemistry or immunofluorescence, mass spectrometry imaging was conducted. Fresh-frozen lymph nodes were sectioned and sublimated with a lipid-ionizing DHB matrix. Lymph nodes were imaged by matrix-assisted laser desorption/ionization (MALDI) mass spectrometry imaging. In this method, the laser ablated a series of 15 μm pixels generating a non-targeted scan of detectable lipids at each pixel. A specific mass was selected and a gradient map generated to display the level of expression across the acquired area. We were able to detect 2 masses matching those of triglyceride (16:1_14:1_18:2; m/z 799.347) and phosphatidylethanolamine (22:0_18:2; m/z 800.327) with a hydrogen adduct (last 2 panels in **Figure 2A** showing the ammonium adduct, and imaging shown in **Figure 2B**). Both lipids appeared to localize to the same areas in the outer regions of each individual skin-draining lymph node, suggesting that the lipids may be draining into the lymph node. Mass spectrometry imaging data alone however did not reveal obvious differences in the lipid location between unirradiated control and UV-irradiated skin-draining lymph nodes.

To more closely interrogate the lymph node cells present in the high lipid areas, we mapped the location of the lipids to the serial section used for immune cell imaging. This revealed that the high lipid areas (as outlined in dotted lines) in the UV lymph nodes were more centrally located than in the control unirradiated lymph nodes (**Figure 2B**). To quantify this, B220 (B cell follicles), CD3 (T cell zones) and CD169 (macrophages in subcapsular sinus) expression within the high lipid “hotspot” areas were calculated as a percentage of the total area of the high lipid region of interest. The UV high lipid areas trended towards decreased CD169^+^ and B220^+^ expression whilst increasing CD3^+^ expression [6 different lymph nodes from 6 individual mice (3 no UV and 3 UV) are shown in **Figure 2C**]. This data indicated that while no specific cell subset was associated with the high lipid regions, UV-induced lipids appear to preferentially locate more towards the T cell areas and away from the subcapsular sinus and B cell follicles.

### 3.3 UV-induced skin-draining lymph node lipids do not alter T cell subsets

**Figure 3:**
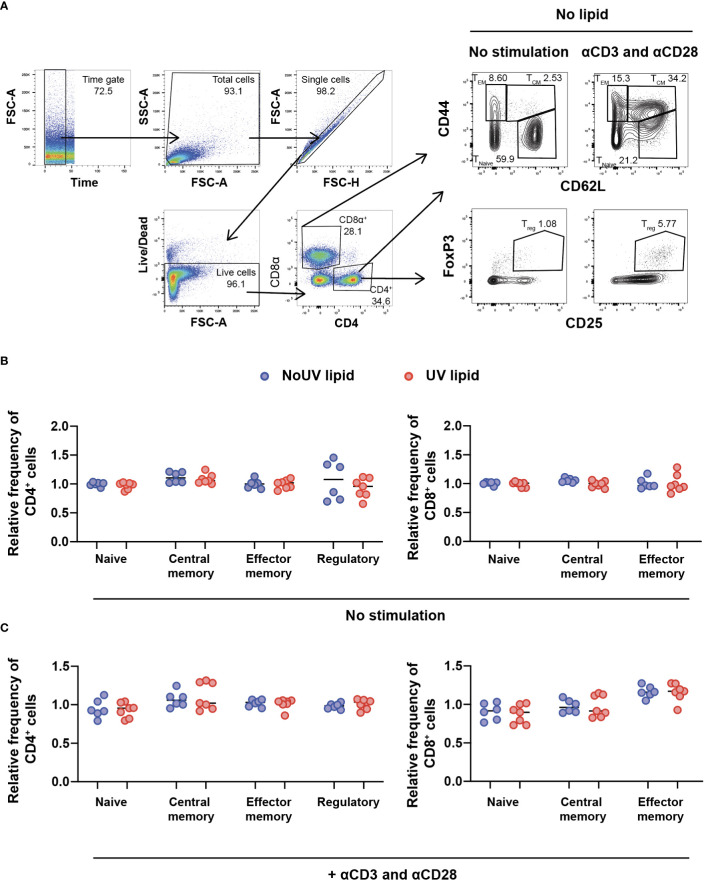
Lipids derived from UV-irradiated skin-draining lymph nodes have no effect on T cell subsets. Skin-draining lymph nodes were collected and lipids extracted. Lipids were then added to untreated skin-draining lymph node cells with or without anti-CD3 and anti-CD28 stimulation. Cells were cultured for 24 hours and stained for flow cytometry analysis. **(A)** Gating strategy for the identification of CD4^+^ and CD8^+^ T cell subsets. CD62L^hi^ CD44^lo^ T cells were defined as naïve, CD62L^hi^ CD44^hi^ T cells as central memory, CD62L^lo^ CD44^hi^ T cells as effector memory and CD4^+^ CD25^+^ FoxP3^+^ cells as regulatory T cells. **(B)** T cell subsets following lipid co-culture without stimulation. **(C)** T cell subsets following lipid co-culture with stimulation. Relative frequency was calculated as a ratio to the no lipid control of each independent experiment. Median and individual mice (lipid donors, n = 6-7) from 2 independent UV-irradiation experiments are shown. Statistics were done by two-tailed unpaired Mann-Whitney test.

Due to the preferential accumulation of UV-induced lipids in the T cell areas of lymph nodes, we next assessed whether UV-induced lipids affected lymphocyte activation and/or differentiation. To address this, we extracted the entire lipid fraction from the skin-draining lymph nodes of unirradiated control and UV-exposed mice. These bulk lipids were added to lymph node cells isolated from untreated skin-draining lymph nodes and incubated for 24 hours before T cell subsets were assessed by flow cytometry. The gating strategy to identify naïve, central memory, effector memory and regulatory T cells is shown in **Figure 3A**. To compare the effect of unirradiated control-derived and UV-derived lipids on T cell activation, the frequencies of subsets were normalized to the no lipid control. No differences were observed in the ability of lipids (from control or irradiated mice) to alter CD4^+^ or CD8^+^ T cells subsets, including Tregs (**Figure 3B**).

The failure of lipids alone to alter T cell subsets could be due to the absence of T cell stimulation. We therefore assessed whether lymph node lipids were able to suppress T cell activation in the presence of anti-CD3 and anti-CD28 antibodies. Similar to what was observed in the absence of T cell stimulation, adding lipids from unirradiated control or UV-irradiated mice did not affect CD4^+^ or CD8^+^ T cell activation in the presence of stimulation (**Figure 3C**). Hence, lipids from skin-draining lymph nodes, whether the skin is exposed to UV or not, do not alter T cell subsets.

### 3.4 UV-induced skin-draining lymph node lipids suppress T cell expansion

**Figure 4:**
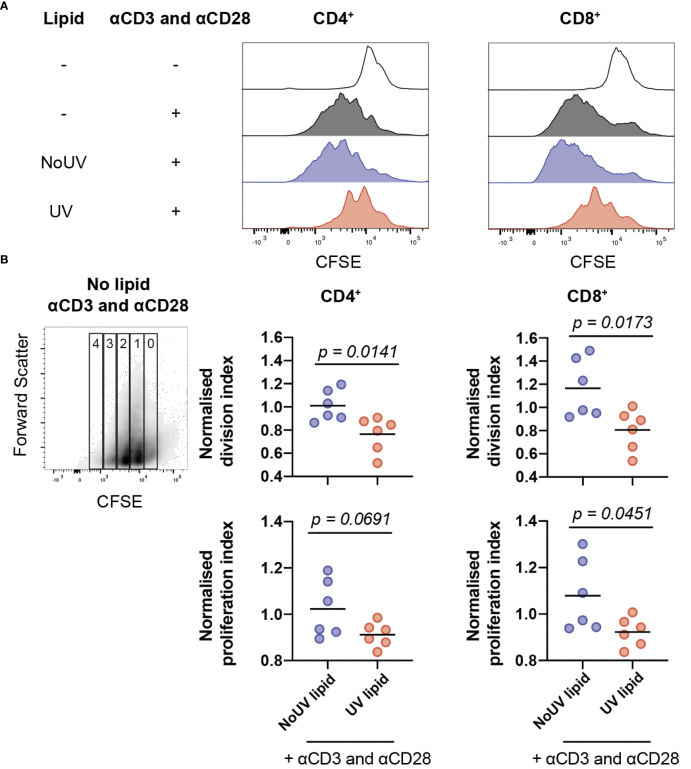
Lipids from UV-irradiated skin-draining lymph nodes suppress T cell proliferation. Skin-draining lymph nodes were collected and lipids extracted. Lipids were then added to CFSE-stained untreated skin-draining lymph node cells with or without anti-CD3 and anti-CD28. Cells were cultured for 72 h and assessed for CFSE expression by flow cytometry. **(A)** Representative histograms of CFSE expression in CD4^+^ and CD8^+^ T cells with and without lipids and/or stimulation. **(B)** Normalized division and proliferation index of CD4^+^ and CD8^+^ T cells with stimulation and either lipids from NoUV or UV mice. Division and proliferation indexes were normalized to the stimulated no lipid control of each independent experiment. Median and individual mice (lipid donors, n = 6) from 2 independent UV irradiation experiments are shown. Statistical analysis was by a two-tailed unpaired Student’s t tests.

Clonal expansion is a requisite event for robust immune responses. Indeed, inhibition of T cell proliferation is a highly effective strategy underpinning the therapeutic success of immune suppressants (37). It was possible, therefore, that lipids from lymph nodes draining UV-exposed skin could be inhibiting T cell proliferation. To assess this, lipids isolated from the skin-draining lymph nodes of unirradiated control and UV-exposed mice were added to CFSE-labelled cells isolated from naïve skin-draining lymph nodes. Anti-CD3 and anti-CD28 antibodies were added to induce proliferation. 72 hours later, the cells were stained and analyzed by flow cytometry. As expected, the addition of anti-CD3 and anti-CD28 successfully caused CD4^+^ and CD8^+^ T cell proliferation. The addition of lipids derived from the skin-draining lymph nodes of mice exposed to UV, but not from unirradiated control mice significantly inhibited both CD4^+^ and CD8^+^ T cell proliferation (**Figure 4A**).

To examine whether the lipids were reducing the number of cells proliferating or reducing the number of divisions each cell undergoes (or both), division index and proliferation index were calculated (38). Lipids from UV-exposed mice significantly decreased the division index for both helper and cytotoxic T cells (**Figure 4B**) indicating that the lipids suppressed the average number of divisions undertaken by all T cells. The proliferation index was also lower for CD8^+^ T cells, but failed to reach statistical significance for CD4^+^ T cells, meaning that once a CD8^+^ T cell commenced proliferation, it underwent fewer divisions in the presence of lipids isolated from the lymph nodes of UV-exposed mice. Together, this indicates that UV-lymph node lipids significantly decrease the number of proliferating cells and, at least for CD8^+^ cytotoxic T cells, reduce the number of divisions undertaken by cells that had commenced proliferation.

### 3.5 Skin-derived large extracellular vesicles rapidly appear in the lymph nodes following UV

**Figure 5:**
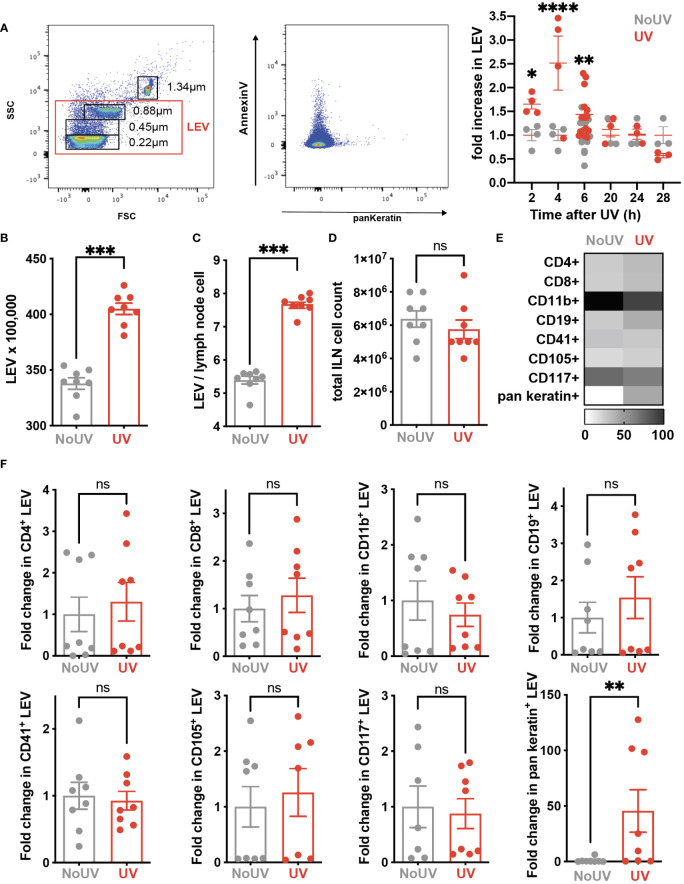
iLN LEV are increased at early but not late timepoints post UV exposure. **(A)** At the times indicated, or at 6h post UV, inguinal lymph nodes (iLN) were isolated and LEV isolated and analysed by flow cytometry. LEVs were gated using size beads (0.22, 0.45, 0.88, 1.34µm) to select particles 0.1-1μm (left plot), with a representative plot showing Keratin^+^ LEVs (right plot). Fold increases in total iLN LEVs at 2, 4, 6, 20 and 24h post UV. At 6h post UV, total iLN LEVs were analyzed as **(B)** x100, 000 and **(C)** per iLN cell, calculated from **(D)** total iLN cell count. **(E)** Heatmap summarises changes in parent LEVs displayed as minimum-maximum percentile scaling where 100 is the maximum for each marker. **(F)** Each point represents an individual mouse UV-exposed (red) or not (grey). ‘Parent’ cell specific LEVs from T cells (CD4^+^ and CD8^+^), B cells (CD19^+^), monocyte/macrophages (CD11b^+^), platelets (CD41^+^), endothelial cells (CD105^+^) mast cells (CD117^+^) and keratinocytes (pan-keratin^+^) were assessed as fold change of absolute numbers x100,000. Statistical analysis was by a 2-way ANOVA with an uncorrected Fisher’s LSD or Mann-Whitney test. *p< 0.05, **p< 0.01, ***p< 0.001, ****p<0.0001; ns, not significant.

Signals generated in UV-exposed skin leads to systemic immune suppression *via* the formation of microvesicles which are submicron (0.1-1μm) large extracellular vesicles (LEV) generated from the budding of cell membranes in response to stressors and danger-signals. LEVs derived from keratinocytes can be readily detected in the skin and plasma of mice and humans exposed to UVB radiation (39). Whether solar-simulated UV-induced skin LEVs find their way to draining lymph nodes has not been investigated before. Since LEVs can transport lipids as both cargo and on the membrane surface (40, 41), it is possible that skin-derived LEVs transport lipids to the skin-draining lymph nodes which could explain how UV alters the lymph node lipidome. To investigate this, groups of mice were exposed to UV before the LEVs in their skin-draining lymph nodes were analyzed by flow cytometry at various times. UV significantly increased the proportion of LEVs in the skin-draining lymph nodes at 2, 4 and 6h after UV (**Figure 5A**). Absolute numbers of LEVs were also increased 6h after UV exposure (**Figure 5B**). This increase preceded hypertrophy of the lymph nodes which typically occurs no earlier than 24h post exposure (**Figures 5C, D**) (8). Detailed flow cytometry analysis found that the only significant UV-induced increase was in keratin^+^ LEV thus confirming their skin origin (**Figure 5E**). Indeed, keratin^+^ LEV in the lymph nodes of control unirradiated mice were almost undetectable (**Figure 5E**). In addition, we found no change in LEV derived from other parent cells including T cells (CD4^+^ and CD8^+^ LEV), B cells (CD19^+^ LEV), monocyte/macrophages (CD11b^+^ LEV), platelets (CD41^+^ LEV), endothelial cells (CD105^+^ LEV) or mast cells (CD117^+^ LEV) (**Figures 5E, F** with representative flow plots shown in **Supplementary Figure 1**). Thus, LEV from UV-exposed skin find their way to secondary lymphoid organs and represent an important way in which peripheral regulatory signals are transmitted to the immune system.

## 4 Discussion

The skin-draining lymph nodes are a major site of UV-induced immune suppression, where the activation of UV-Tregs, UV-Bregs and sequestration of naïve and central memory T cells occurs. Here we have demonstrated that UV upregulates 6 unique, previously undescribed UV-induced lipids, mostly in the T cell zones of lymph nodes, and that some of these lipids can suppress the expansion of T cells.

We previously showed that exposure to UVB prior to contact sensitization inhibits the expansion of effector T cells in the skin-draining lymph nodes (42). Until now, the mechanism behind this suppression was not known. There was no evidence for any of the described processes including prostaglandin E2 (PGE_2_), pyrimidine dimers, *cis*-urocanic acid, reactive oxygen species, or the generation of functional Tregs (42, 43), to be responsible for this suppression. Here we show that lipids isolated from UV-irradiated skin-draining lymph nodes suppress T cell expansion. The lipid extraction method we used has been previously established as resulting in very minimal non-lipid material being extracted (44) and has been used in other similar lipid-tissue culture experiments (45), giving us confidence that the immune suppressive effects observed were indeed lipid-mediated. This suggests that UV-induced lipids in the local draining lymph nodes are at least in part responsible for a dampened T cell response post-UV exposure.

Our lipidomic analysis identified 2 acylcarnitines, a phosphatidylcholine, a diglyceride, a triglyceride and a phosphatidylethanolamine as being significantly increased in the skin-draining lymph nodes of UV-exposed mice. Previous studies have identified the lipids PGE2, PAF and arachidonic acid as being involved in UV-immune suppression (28). PGE2 is unlikely to be detected using untargeted mass spectrometry as it is too low in abundance. And due to its extremely short half-life, PAF is unlikely to be detectable under the experimental conditions used. However, the increased levels of acylcarnitine (20:4) we observed (**Figure 2A**) is a close surrogate for increased arachidonic acid as arachidonic acid is released from lipids that contain the arachidonyl fatty acid chain (20:4) in response to cell stimuli, usually associated with activation of phospholipase A2 (the enzyme that cleaves the 20:4 fatty acid from the lipid).

Whilst there is limited knowledge on how the specific lipids we identified affect cell-mediated immunity, the changes in acylcarnitines, diglycerides and triglycerides suggest that UV alters fatty acid metabolism. Indeed this is known to occur in the skin following UV exposure, whereby the expression of genes related to lipid synthesis are decreased (23, 24). Our group has also shown that UV exposure on the skin significantly increases total liver triglycerides (46). The possibility of UV modulating fatty acid metabolism is of particular importance as naïve T cells, but not effector T cells, use fatty acid oxidation and have high levels of acylcarnitine molecules (47, 48). Furthermore, regulatory T cells also use lipid oxidation to sustain proliferation (17–19). Since an increase in naïve T cells (16) and regulatory T cells (49) occurs in the skin-draining lymph nodes following UV irradiation, this potential change in lipid metabolism may be an additional mechanism of maintaining naïve and regulatory T cells numbers in the lymph nodes.

Whilst the fragmentation data allowed for the identification of the lipid headgroup and acyl chains, the location of double bonds within the fatty acyl chain and the position of fatty acids on the glycerol headgroup cannot be distinguished by LC-MS/MS with electrospray ionization, as used for this study. This means that the specific lipids identified in this study cannot be synthesized currently as the exact structure is unknown. As the molecular structure affects the bioactivity of the lipid, this needs to be ascertained before functional studies can be conducted. Additionally, commercially available lipids typically contain identical fatty acids meaning that the UV-lipids identified in this study cannot be purchased, restricting the functional assays possible to interrogate whether the specific increases in lipids we have observed are responsible for inhibiting T cell expansion.

Visualization of UV-altered lipids using imaging mass spectrometry in combination with imaging mass cytometry was a powerful interrogation tool which revealed that lipids with masses close to triglyceride and phosphatidylethanolamine were found preferentially in T cell areas in the lymph nodes and further away from B cell follicles. In this study, mass spectrometry imaging was critical as lipids cannot be imaged by traditional immunohistochemical or immunofluorescent methods. However, mass spectrometry imaging does have some limitations. Mass accuracy is particularly important as it allows for the accurate identification of the lipid ion. However, the mass accuracies varied for the instrument used for lipidomic analysis (mass accuracy of 1 ppm) and imaging mass spectrometry (mass accuracy of ± 30 to 120 mDa). Since the imaging mass spectrometer had low mass accuracy and no fragmentation was done to ascertain the exact lipid identity, we cannot be certain that the same lipids identified in the lipidomic studies were imaged. Similarly, without an exact lipid identity, we are forced to assume that the imaged lipids were independent of each other and not isotopic ions (the same compound but with a series of ions differing by one *m/z* unit). In addition, the imaging mass spectrometer has lower sensitivity in comparison to the quadrupole-orbitrap mass spectrometer used for our lipidomics studies. This resulted in some of the low-abundance UV-induced lipids not being imaged.

This study has highlighted the ability of UV-induced lipids to suppress T cell proliferation in the skin-draining lymph nodes. A number of potential lipid candidates responsible for the suppression have been identified. Simultaneous sequestration of naïve and central memory T cells in the lymph nodes (16) will maximise the chances that the immune suppressive lipids we have discovered can influence T cell fate. The current limitations of lipid imaging resolution means that it is not yet known whether the associated cells are producing the lipid or are affected by the lipid. The conspicuous location of the lipids around the outer regions of the lymph nodes suggests that the lipids may be draining into the lymph nodes from the irradiated skin. Alternatively, exposure of the skin to UV, which enlarges dermal lymphatic vessels (50) and increases vascular flow, may result in increased drainage of cutaneous UV-induced lipids. This hypothesis would be consistent with free fatty acids and triglycerides being depleted in the epidermis following UV exposure (23). An alternative possibility that we considered and tested is that UV-induced skin lipids reach the lymph nodes packaged within large extracellular vesicles (LEV). UV induces LEV formation by keratinocytes in a PAF-dependent manner (51, 52) and in humans, LEV numbers within the skin and plasma (flow cytometrically identified by their surface expression of calcium-sensing receptors) are significantly increased post-UVB exposure (39). Our data shows that the same immune suppressive dose of solar simulated UV significantly increases the number of keratin-expressing LEVs in the local-draining lymph nodes 6 hours following exposure. Increases in skin-derived mast cells are not detectable in lymph nodes until 24h after UV exposure (8) so these events would appear to be distinct. To our knowledge this is the first-time antibodies to keratins have been used to identify skin-derived LEV. This approach is supported by proteomic data confirming the expression of keratins in LEVs (53). Showing that skin-derived LEVs are the source of the T cell-suppressing lipids in draining lymph nodes awaits next generation flow cytometers that can sort keratin+ LEVs for lipidomic analysis. In the meantime, studies inhibiting UV production of LEVs with topical acid sphingomyelinase inhibitors like imipramine could be performed. Indeed, this pharmacological strategy has already been shown to be effective in mice (39) and is currently being trialed in humans (NCT04520217).

Precisely how UV alters systemic immune responses is not well known. This is important if we are to harness the beneficial effects of UV to prevent and treat non-skin diseases like multiple sclerosis (5). The mechanisms appear to be different to that which mediate local immune suppression at the irradiated site. The release of skin-LEVs containing PAF (39) and alterations to S1P in lymph node nodes (16) are novel and major ways in which UV modulates distant, non-skin immune responses. Establishing a lymph node lipidome that suppresses T cell proliferation appears to be another.

## Data availability statement

The original contributions presented in the study are included in the article/**Supplementary Material**. Further inquiries can be directed to the corresponding author.

## Ethics statement

The animal study was reviewed and approved by The University of Sydney Animal Ethics Committee.

## Author contributions

BT helped design and performed most of the experiments, analyzed and interpreted most of the data, prepared the majority of the figures and wrote the first draft of the manuscript. AF helped design and performed some of the experiments, analyzed and interpreted some of the data, and prepared some of the figures. YK and AD helped design, perform some of the experiments and interpret the results. GG helped design some of the experiments and interpret some of the data. SB supervised BT and AF, designed the experiments, analyzed and interpreted the data, finalized the figures and wrote the final draft of the manuscript. All authors contributed to the article and approved the submitted version.
