# Supplementary figures and images for: Exposure to solar ultraviolet radiation establishes a novel immune suppressive lipidome in skin-draining lymph nodes
Source: Front Immunol. 2023 Jan 20;13:1045731. doi: 10.3389/fimmu.2022.1045731 (PMC9895826; doi:10.3389/fimmu.2022.1045731)

Supplementary Figure 1

AnnexinV

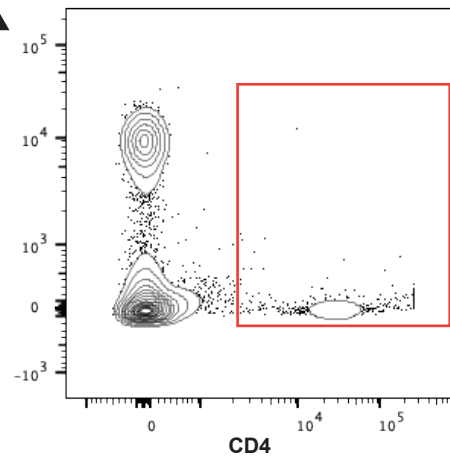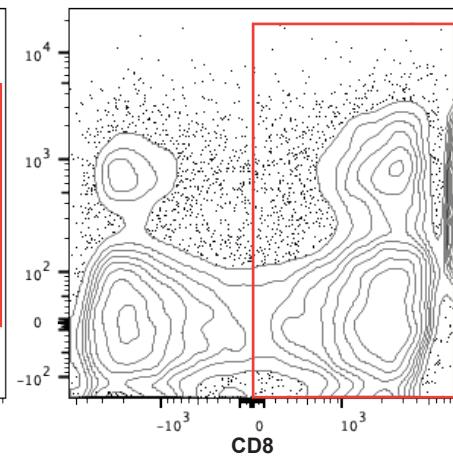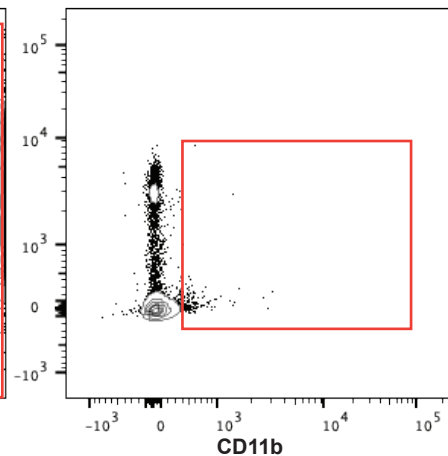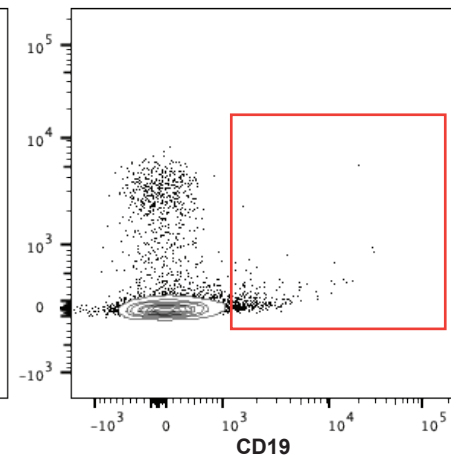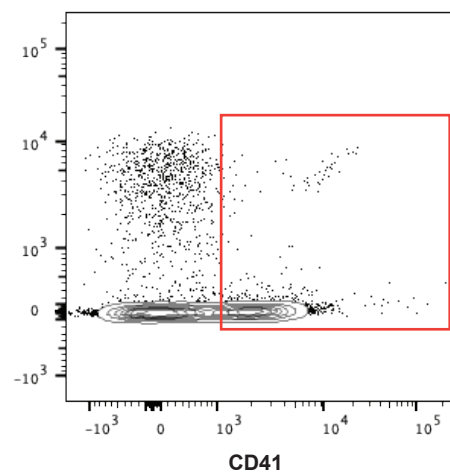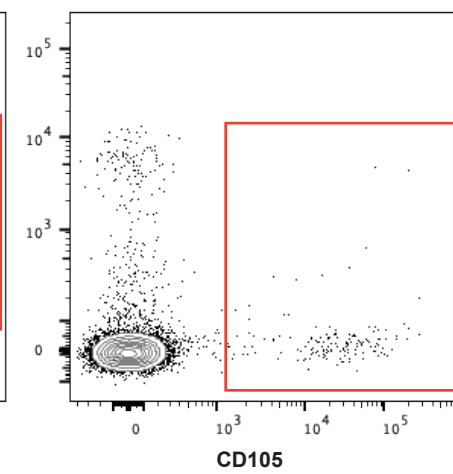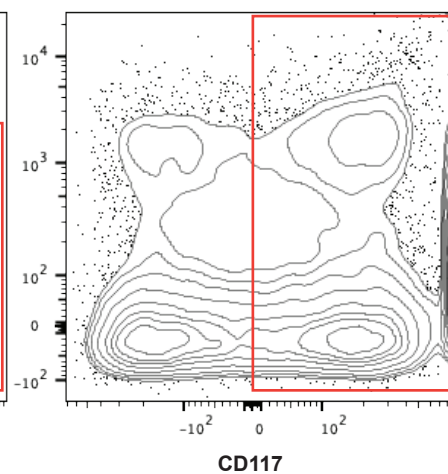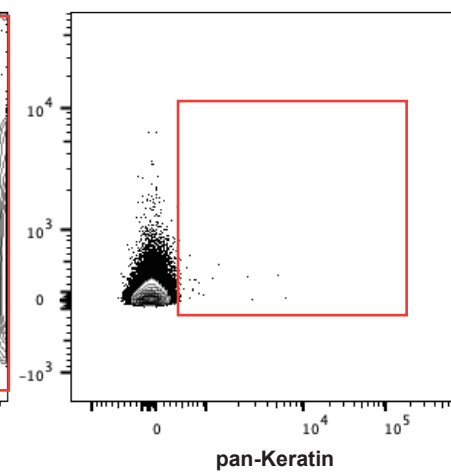

Supplement: Supplementary file 1 [file Image_1.pdf]
